# Inhibition of Interleukin-1 Receptor-Associated Kinases 1/4, Increases Gene Expression and Serum Level of Adiponectin in Mouse Model of Insulin Resistance

**DOI:** 10.22088/IJMCM.BUMS.7.3.185

**Published:** 2018-10-14

**Authors:** Athena Rajaie, Mostafa Allahyari, Mahdieh Nazari-Robati, Hossein Fallah

**Affiliations:** Department of Clinical Biochemistry, Afzalipour School of Medicine, Kerman University of Medical Sciences, Kerman, Iran.

**Keywords:** Insulin resistance, inflammation, adiponectin, IRAK inhibitor

## Abstract

Insulin resistance is a feature of most patients with type 2 diabetes mellitus. Epidemiological evidence suggest a correlation between inflammation and insulin resistant states such as obesity, but the underlying mechanisms are largely unknown. Interleukin-1 receptor-associated kinases (IRAK) play a central role in inflammatory responses by regulating the expression of various inflammatory genes in immune cells. This study was aimed to investigate the effect of IRAK inhibitor on gene transcription and serum concentration of adiponectin in insulin-resistant mice. Experimental mice were randomly divided into 6 groups: the healthy control group was fed a regular chow diet while other groups were fed with a high-fat diet for 12 weeks. After this period, the animals were treated with IRAK inhibitor, pioglitazone, both IRAK and pioglitazone, and DMSO, for two weeks. Adiponectin gene expression level was analyzed by real-time PCR. Additionally, serum adiponectin levels were measured by ELISA. Homeostasis model assessment-adiponectin (HOMA-AD) as an insulin sensitivity index was calculated. IRAK inhibitor and pioglitazone increased significantly the expression of adiponectin gene. Also, adiponectin concentration in the control group (9.67±1.1 μg/ml) increased to 25.34±2.04 μg/ml in pioglitazone treatment group. IRAK inhibitor also increased adiponectin concentration (18.24±1.53 μg/ml) but did not show a synergistic effect with pioglitazone when administered simultaneously (26.66±2.5 μg/ml). HOMA-AD was 0.33±0.04 in pioglitazone treated group, 0.6±0.13 in IRAK inhibitor group, and 0.31±0.03 in animals that received IRAKi and pioglitazone. Our findings suggest that increased adiponectin secretion from adipose tissue mediated by IRAK inhibitor may increase the insulin sensitivity in an animal model of insulin resistance.

Insulin resistance (IR) is a complicated condition in which three primary metabolic tissues that are sensitive to insulin; skeletal muscle, liver, and white adipose tissue (WAT) become less sensitive to insulin and its downstream metabolic actions under normal serum glucose concentrations (1). IR is the condition in which a cell, tissue, or organism fails to respond appropriately to a given dose of insulin (2).

IR accompanies a wide range of pathological conditions, including obesity, lipodystrophy, sepsis, steroid use, growth hormone excess, polycystic ovarian syndrome, cancer, neuro-degenerative disease, hypertension, hyperglycemia, and metabolic syndrome (1) and even some physiological conditions, such as pregnancy (2).

Obesity, characterized as a state of chronic low-grade inflammation caused by over- nutrition, is a major cause of decreased insulin sensitivity, which makes obesity a major risk factor for IR (1).

Factors released from adipose tissue that could contribute to the development of IR and B-cell dysfunction, include tumor necrosis factor α (TNF-α), free fatty acids (FFAs), adiponectin, resistin, leptin, agonists of the peroxisome proliferator-activated receptor γ (PPARγ) (3), interleukin (IL)-1β, IL-6, monocyte chemoattractant protein-1 (MCP-1), nuclear factor kappa B (NFκ-B), c-Jun N-terminal kinase (JNK), macrophage, high-sensitivity C-reactive protein (hs-CRP), the JAK-STAT signaling pathway (1).

Adiponectin, an adipocyte-specific secretory protein carrying 244 amino acids with 18 signal residues (4) is an adipokine that is specifically and abundantly expressed in adipose tissue, and directly sensitizes the body to insulin. The adiponectin receptors, AdipoR1 and AdipoR2, which mediate the antidiabetic metabolic actions of adiponectin, have been cloned, and are downregulated in obesity-linked IR (5).

Adiponectin, the adipocyte hormone with the highest plasma concentration, is considered a modulator of carbohydrate and lipid metabolism and a marker of insulin sensitivity. Although mainly produced in adipose tissue, serum adiponectin concentrations are negatively correlated with the amount of visceral adiposity (6).

Numerous clinical studies demonstrated an inverse relationship between serum adiponectin levels and overproduction of pro-inflammatory markers such as TNF-α and CRP (7). Given that IL-6 is pro-inflammatory, it is widely accepted that like TNF-α, IL-6 negatively impacts obesity-induced IR (8).

Thiazolidinediones, including pioglitazone, constitute a new class of oral antidiabetic drugs that are widely used as insulin-sensitizing agents through the activation of PPAR-γ, thereby regulating the transcription of specific genes involved in adipogenesis and IR (9).

Inflammation plays an important role in the development of IR via various cytokines and molecular pathways, and may therefore be targeted with appropriate interventions to prevent IR (1). Interleukin 1 receptor-associated kinase 1(IRAK1) mediates pro-inflammatory signaling via IL-1 receptor/toll-like receptors, which may contribute to IR. IL-1-R and toll-like receptors interact with MyD88 to activate IRAK-4 which phosphorylates and activates IRAK-1. Downstream from IRAK-1, TNF receptor associated factor 6 (TRAF6) interacts with Mitogen-activated protein kinase kinase kinase 7 (MAP3K7) to activate the IκB kinase (IKK) complex leading to phosphorylation of IκBα that then dissociates from NF-κB. NF-κB enters the nucleus to promote the transcription of many pro-inflammatory genes including interferons and cytokines, such as IL-1 and TNF-α (10). IRAK-1 phosphorylate insulin receptor substrate 1 (IRS-1) at Ser24, which impairs binding of IRS-1 to phosphatidylinositol 3-kinase (PI3K), thereby impairing metabolic actions of insulin including insulin-stimulated translocation of glucose transporter type 4 (GLUT4) (1).

IRAK-1, which impairs insulin signaling, thereby induces IR. Understanding the detailed mechanisms of this cross-talk between pro-inflammatory signaling pathways and insulin signaling pathways may help to improve the diagnosis of pre-diabetic states, and also suggest new strategies to treat diabetes (10). Potent and selective small molecule inhibitors targeting IRAK-4 kinase activity are currently being pursued as potential therapeutics for autoimmune and infla-mmatory diseases (11).

IRAK inhibitor can suppress the inflammation pathways and may therefore increase adiponectin expression and secretion by decreasing the level of pro-inflammatory cytokines. Thereupon, we aimed to evaluate the adiponectin gene expression level under treatment with IRAK inhibitor.

## Materials and methods

**Animals**

 In this experimental study, male C57BL/6 mice, (mean weight 19 g, 6 weeks-of-age) were obtained from the animal house of Pasteur Institute of Iran (IPI). The Ethics Committee of Kerman University of Medical Sciences approved the study procedure. The animals were maintained in a temperature-controlled room (22°C) on a 12-h light-dark cycle. One week after arrival, mice were randomly divided into 6 groups (n= 8 each) and 5 groups were fed high-fat diet while one group received continuous feeding of a normal diet for 12 weeks. High-fat diet was composed of 365 g regular chow, 250 g casein, 310 g fat, 60 g minerals and vitamins, 60 g cholesterol, 3 g methionine, and 2 g cholic acid per Kg (12). 

**Treatment**

After 12 weeks, the animals were treated for two additional weeks as follows: healthy control group received normal diet without any drug; control group received high-fat diet without any drug; IRAK inhibitor (IRAKi) group received high-fat diet with intra peritoneal (i.p.) injection of 2.12 mg/kg IRAKi 3 times weekly (13). IRAKi was diluted in DMSO (5 mM) and further dissolved in sterile PBS (pH 7.2). Pioglitazone (Pio) group received high-fat diet and intra gastric infection of pioglitazone (10 mg/kg/day) (9). IRAKi+Pio group received high-fat diet with pioglitazone and IRAKi as mentioned above. The vehicle group was fed with high-fat diet and DMSO.

**Blood and tissue collection**

At the end of the treatment period (2 weeks), animals were fasted for 12 h overnight. A separate group of animals was killed by cardiac puncture under terminal anesthesia induced by diethyl ether (14). Visceral adipose tissue samples were harvested. All samples were flash frozen in liquid nitrogen and subsequently stored at -75 ℃ until evaluation.

M**easurement of serum adiponectin**

Serum samples were separated from blood, by centrifuge at 3000 rpm for 10 min at 4 ℃. Adiponectin level was determined with a double-antibody radioimmunoassay (ZellBio GmbH kit, Germany). Homeostasis model assessment of adiponectin (HOMA-ID) was calculated using the equation: [(insulin (U/ml) × glucose (mmol/l))/22.5×adiponectin (μg/ml)] (15).

**Total RNA extraction**

Total RNA was extracted from visceral adipose tissue samples using a RNeasy Mini Kit (Favorgen Biotech Corp, Taiwan) incorporating DNase digestion. Extracted total RNA was quantified using a NanoDrop spectrophotometer (ND-1000 nanodrop). RNA quality was assessed by ethidium bromide staining of 18S and 28S ribosomal RNA after electrophoresis on 1.5% agarose gel. Complementary cDNA templates for real-time PCR assays were prepared from Moloney Murine Leukemia Virus Reverse Transcriptase (M-MLV RT), oligodT primer (Favorgen Biotech Corp, Taiwan) and total RNA (2.5 µg). 

**Real Time PCR**

Real-time quantitative PCR amplification was conducted with the mic Biomolecular systems (BMS, Australia) using YTA Super SYBR Green qPCR MasterMix 2X (Favorgen Biotech Corp, Taiwan) according to the protocol recommended by the manufacturer. PCR was run as follows: initial activation of Taq DNA polymerase at 94 ℃ for 3 min, 5 s at 95 ℃ for denaturing, 5 s at 55 ℃ for annealing, 10 s at 72 ℃ for elongation, and 40 PCR cycles were performed. All experiments were run in duplicate and non-template controls and dissociation curves were used to detect primer–dimer conformation and nonspecific amplification. According to the similarity of PCR efficiency for adiponectin and *GAPDH*, difference in gene expression was calculated by 2^-∆∆CT ^method.

Primer sequences are described in Table 1 (Pishgam Biotech Co, Iran).

**Statistical analysis:**

All data were analyzed using SPSS software, Version 22. For determination of difference between groups, nonparametric Kruskal-Wallis test was used. All data were presented as Mean ± SE. P< 0.05 was considered statistically significant. For determination of correlation between HOMA-IR and adiponectin, Spearman's Rho test was used.

**Table 1:** Sequence of primers

**Gene**	**Primers 5’ → 3’**	**Amplicon size (bp)**
Adiponectin	F:ATGGCAGAGATGGCACTCCT	162
	R:CATAAGCGGCTTCTCCAGGC	
*GAPDH*	F:ACCATCTTCCAGGAGCGAGA	
	R:GAAGGGGCGGAGATGATGAC	152

**Fig. 1:**
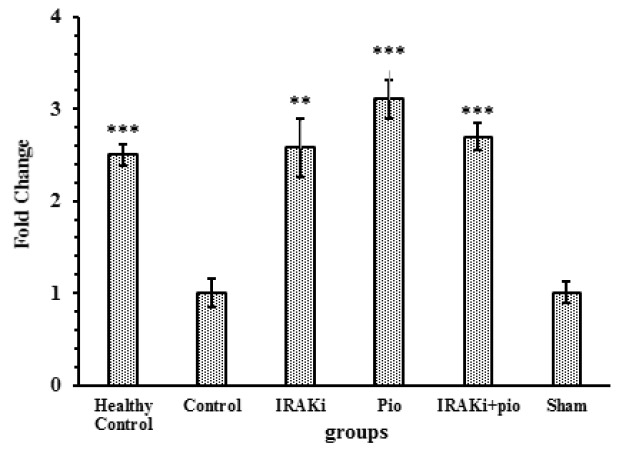
Transcription level of adiponectin gene. Each IRAK inhibitor or pioglitazone increased the expression level of adiponectin gene in high-fat diet mice. IRAKi: IRAK inhibitor; pio: pioglitazone. *** P< 0.0001, ** P<0.01, * P<0.05 in comparison with control group

## Results

**Transcription level of adiponectin gene**

RT-qPCR results showed that IRAK inhibitor significantly increased the expression of adiponectin gene (P<0.0001). Also, pioglitazone increased adiponectin gene expression (P=0.0027), although these agents did not show any incremental effect on each other. Results of adiponectin gene expression evaluation are summarized in figure 1.

**Adiponectin serum concentration**

**Fig. 2:**
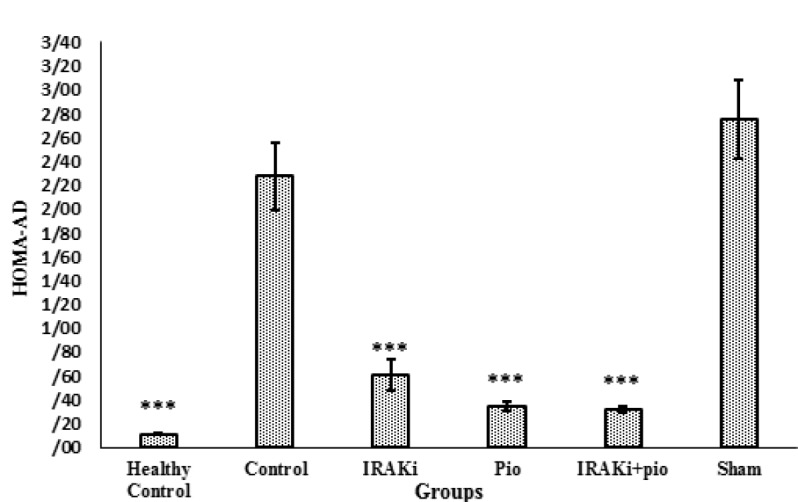
Serum concentration of adiponectin. IRAK inhibitor or pioglitazone increased the serum concentration level of adiponectin in high- fat diet mice. IRAKi: IRAK inhibitor; pio: pioglitazone.*** P< 0.0001, ** P< 0.01, * P< 0.05 in comparison with control group

High-fat diet resulted in a decrease of serum adiponectin concentration (9.67±1.1 μg/ml) in comparison with healthy control group (24.02±1.24 μg/ml) that received normal diet. However, pioglitazone treatment increased adiponectin concentration to 25.34±2.04 μg/ml. IRAK inhibitor also increased adiponectin concentration (18.24±1.53 μg/ml) but could not exert a synergistic effect on pioglitazone when administred simultaneously (26.66±2.5 μg/ml) (figure 2).

**Fig. 3:**
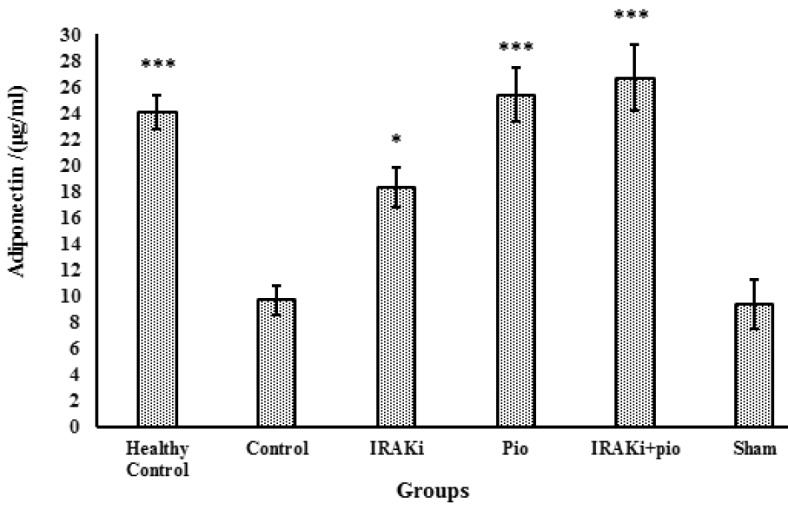
Effects of IRAKi on HOMA-AD..IRAK inhibitor or pioglitazone decreased HOMA-AD in high-fat diet mice. IRAKi: IRAK inhibitor; pio: pioglitazone. *** P< 0.0001, ** P< 0.01, * P< 0.05 in comparison with control group

**Fig. 4:**
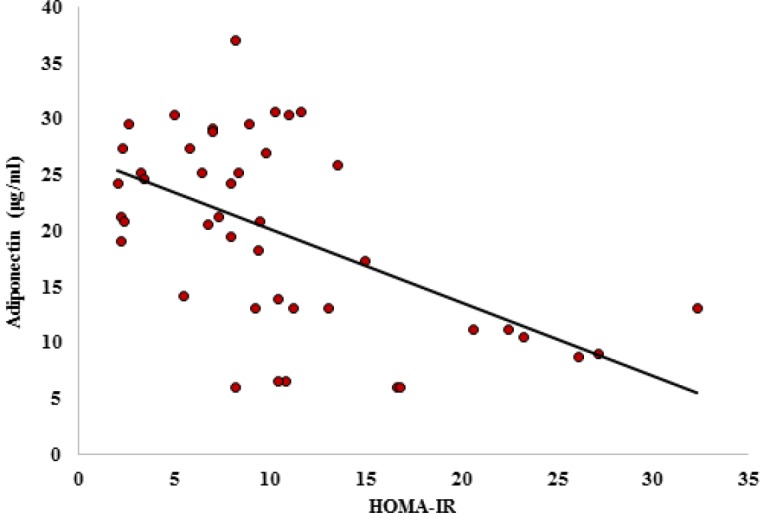
Correlation between HOMA-IR and adiponectin. Correlation coefficient= -0.527; P< 0.0001

**HOMA-AD: Index of insulin sensitivity**

As shown in figure 3, HOMA-AD was 0.33±0.04 in pioglitazone treated group, 0.6±0.13 in IRAK inhibitor group, and 0.31±0.03 in animals that received IRAKi and pioglitazone. These results were significantly lower than the control group (2.27±0.28, P<0.0001). HOMA-AD in healthy animals was 0.10±0.007 that is indicative of high insulin sensitivity of these animals.

**HOMA-IR and adiponectin correlation**

There was a significant (P<0.0001) and negative correlation (correlation coefficient=-0.527) between adiponectin concentration and HOMA-IR (figure 4) shows this correlation.

## Discussion

In obesity state, adipose tissue is developed and its macrophages are increased. Low-grade inflammation increases blood free fatty acid levels which results from obesity, and can induce IR and type 2 diabetes (16, 17). Obesity also is related to a decrease of adiponectin concentration. Adiponectin is an insulin sensitizer adipokine (18). IRAK inhibitor, as an interference in inflammation pathways, can increase insulin sensitivity. Therefore, we evaluated the effects of this factor in comparison with pioglitazone on adiponectin gene expression at mRNA and protein levels.

Our results showed that IRAK inhibitor and pioglitazone increased the transcription level of adiponectin. Also, adiponectin concentration increased in serum of insulin-resistant mice by IRAK inhibitor and pioglitazone.

In Toll-Like Receptor signaling pathways which are activated by lipids, lipoproteins, lipopolysaccharides, and proteins, MYD88 is complexed with IRAK4 and IRAK1. IRAK4 activates IRAK1. Activated IRAK1 is released from the complex and through a specific signaling cascade, finally activates NF-κB, a key factor in inflammation response, which increases the transcription of some pro-inflammatory cytokines (17, 19). IRAK inhibitor by suppression of this signaling pathway can induce anti-inflammatory functions (11) and decrease IR which results from inflammation.

There is a negative correlation between BMI and adiponectin concentration so obesity along with its related inflammation decreases adiponectin production (20, 21). Adiponectin production is controlled by several factors. Pro-inflammatory cytokines that increased in IR, control negatively the production of adiponectin. In contrast, PPARγ increases the expression of adiponectin in adipose tissue (22). This is compatible with our results that pioglitazone as a PPARγ agonist and IRAK inhibitor as an anti-inflammatory factor increased the level of adiponectin.

Overexpression of IRAK can induce inflammation and decrease adiponectin level. So, it is not surprising that IRAK inhibitor increased adiponectin concentration (23). However, deletion of the IRAK1 gene can increase insulin sensitivity without any effect on adiponectin level (10).

HOMA-AD is an insulin sensitivity index, and our results showed that IRAK inhibitor significantly increased the insulin sensitivity with an efficiency completely comparable with pioglitazone. Also, we showed a significant and negative correlation between HOMA-IR and adiponectin levels, suggesting that adiponectin plays a critical role in tolerance of glucose and increase of sensitivity to insulin. Furthermore, adiponectin can act as a predictorof IR, so that increase in adiponectin level, decreases IR, and increase in IR is accompanied by adiponectin decrease.

In our study, pioglitazone and IRAK inhibitor did not show any synergistic or antagonist effect on each other, suggesting that each of them exert their effects in an independent manner. Pioglitazone as a PPARγ agonist increases the expression of adiponectin while IRAK inhibitor increases the adiponectin level by suppression of pro-inflammatory cytokines (22, 24, 25).

Adiponectin through increasing of fatty acid oxidation and glucose uptake, increases insulin sensitivity (26). In addition, adiponectin has anti-inflammatory activity and decreases NF.κB dependent inflammation (18).

In conclusion, IRAK inhibitor increases adiponectin production and secretion from adipocytes. Adiponectin is an insulin sensitizer adipokine and for this reason, it is possible that IR decrease by IRAKi is mediated by adiponectin gene overexpression. IRAK inhibitor efficacy in reducing HOMA-AD is fully comparable with pioglitazone and further studies may lead to its introduction as a new antidiabetic agent.

## Conflict of interest

Authors declare no conflict of interest.
